# The transition from noncoded to coded protein synthesis: did coding mRNAs arise from stability-enhancing binding partners to tRNA?

**DOI:** 10.1186/1745-6150-5-16

**Published:** 2010-04-09

**Authors:** Harold S Bernhardt, Warren P Tate

**Affiliations:** 1 Department of Biochemistry, Otago School of Medical Sciences, University of Otago, Dunedin, New Zealand

## Abstract

**Background:**

Understanding the origin of protein synthesis has been notoriously difficult. We have taken as a starting premise Wolf and Koonin's view that "evolution of the translation system is envisaged to occur in a compartmentalized ensemble of replicating, co-selected RNA segments, i.e., in an RNA world containing ribozymes with versatile activities".

**Presentation of the hypothesis:**

We propose that coded protein synthesis arose from a noncoded process in an RNA world as a natural consequence of the accumulation of a range of early tRNAs and their serendipitous RNA binding partners. We propose that, initially, RNA molecules with 3' CCA termini that could be aminoacylated by ribozymes, together with an ancestral peptidyl transferase ribozyme, produced small peptides with random or repetitive sequences. Our concept is that the first tRNA arose in this context from the ligation of two RNA hairpins and could be similarly aminoacylated at its 3' end to become a substrate for peptidyl transfer catalyzed by the ancestral ribozyme. Within this RNA world we hypothesize that proto-mRNAs appeared first simply as serendipitous binding partners, forming complementary base pair interactions with the anticodon loops of tRNA pairs. Initially this may have enhanced stability of the paired tRNA molecules so they were held together in close proximity, better positioning the 3' CCA termini for peptidyl transfer and enhancing the rate of peptide synthesis. If there were a selective advantage for the ensemble through the peptide products synthesized, it would provide a natural pathway for the evolution of a coding system with the expansion of a cohort of different tRNAs and their binding partners. The whole process could have occurred quite unremarkably for such a profound acquisition.

**Testing the hypothesis:**

It should be possible to test the different parts of our model using the isolated contemporary 50S ribosomal subunit initially, and then with RNAs transcribed *in vitro *together with a minimal set of ribosomal proteins that are required today to support protein synthesis.

**Implications of the hypothesis:**

This model proposes that genetic coding arose *de novo *from complementary base pair interactions between tRNAs and single-stranded RNAs present in the immediate environment.

**Reviewers:**

This article was reviewed by Eugene Koonin, Rob Knight and Berthold Kastner (nominated by Laura Landweber).

## Background

Wolf and Koonin [1] have described the origin of the translation system as "arguably, the central and the hardest problem in the study of the origin of life, and one of the hardest in all evolutionary biology". This is due not only to the overwhelming complexity of contemporary protein synthesis with its large number of components and the large size of some of these components, and the extreme unlikelihood that these could have all arisen simultaneously, but also to the fact that evolution is not goal-driven: "Since evolution has no foresight, the translation system could not evolve in the RNA World as the result of selection for protein synthesis and must have been a by-product of evolution driven by selection for another function" [1]. In the most well known version of this idea, Weiss and Cherry [2], Gordon [3] and Penny and colleagues [4,5] have proposed that the small ribosomal subunit evolved from an RNA replicase/triplicase that replicated RNAs using the anticodon triplets of tRNAs rather than mononucleotides, in a process possibly driven energetically by concomitant peptide synthesis [2,4,5]. Originally proposed as a model for the origin of translocation [2], the triplicase hypothesis has the benefit of satisfying the perceived need for a prior function for the proto-ribosome, plus the added bonus of explaining the absence of evidence for an RNA replicase in contemporary biology, presumably essential to the existence of an RNA world [5]. One piece of evidence given in support of the model [3] is the existence of an *E. coli *anticodon nuclease which is able to cleave tRNA immediately 5' to the anticodon [6]. However, as Wolf and Koonin [1] point out, a triplicase/protoribosome would have to be a "tremendously advanced, complex RNA machine", concluding that, "the triplicase might not be the most likely solution to the origin of translation problem".

In this context, the argument that evolution is not goal-driven ignores the possibility that the evolution of coded protein synthesis may have been driven by the accumulation of incremental advances which led initially from the synthesis of random short peptides by a noncoded process through to the eventual production of long complex proteins by a system of coded protein synthesis. This view holds that the contemporary (sometimes very large) components have evolved from smaller molecules that possessed, if not all, at least some of their current functions. Thus, the larger problem can be distilled down to a smaller problem: how did the two main functions of the contemporary ribosome, peptide synthesis and decoding, arise? As W. Ford Doolittle has stated, of these two, peptide synthesis presumably came first, as without it there would be nothing to be coded (comment in [7]). Peptide synthesis is a function of the large ribosomal subunit RNA of the contemporary ribosome, so key to this discussion is the origin of this structure. Maizels and Weiner [8], Bokov and Steinberg [9] and Yonath and associates [10] have all proposed that the large ribosomal subunit RNA arose from the duplication of a ribozyme able to bind an RNA hairpin possessing an aminoacylated 3' CCA terminus. Bokov and Steinberg [9] have deconstructed the large ribosomal subunit RNA based on an analysis of the distribution of tertiary structure A-minor interactions, in which unpaired nucleotide bases (usually adenines) interact with a double helix [11]. Reasoning that the double helix component is stable in the *absence *of the unpaired adenosines but not *vice versa*, they argue that the double helices must have evolved first, allowing them to define the ancestral components of the large ribosomal subunit RNA. When this was done, a region of Domain V of the large subunit RNA was identified consisting of two consecutive 110-nucleotide fragments having almost identical secondary and tertiary structure, corresponding to the A and P sites of the peptidyl transferase centre (PTC) where peptide synthesis occurs in the contemporary ribosome. Bokov and Steinberg's findings are in agreement with those of Yonath and colleagues [12] who made the observation that the "backbone folds [of the A and P sites of the PTC], irrespective of the nucleotide sequences, are related by pseudo two fold symmetry". The ribose-phosphate backbones of the two proposed duplicated segments map onto each other extremely closely.

Smith *et al. *[7] have also presented a theory for the origin of the ribosome that supports the large ribosomal subunit arising first. However, their argument is based largely on the fact that, unlike the case of the peptidyl transfer centre, there is no single self-folding RNA segment comprising the decoding site of the small subunit; why this should infer a more recent origin is not intrinsically obvious, as the small subunit RNA had to evolve at *some *time. It would be interesting to repeat the deconstruction performed on the large subunit RNA (based on the A-minor interaction) [9] for the small subunit RNA. However, if the two subunits originally evolved in isolation (with the regions of interaction evolving at a later stage), such a chronology may not be informative for how the early parts of the two evolutionary histories relate.

In the absence of the small ribosomal subunit (which is responsible for decoding in the contemporary ribosome), peptide synthesis catalysed by the ancestral peptidyl transferase ribozyme would necessarily have been *non*coded. The evolution of coded protein synthesis from such an earlier noncoded system has been proposed by a number of researchers, including Orgel [13], de Duve [14-16], Schimmel [17,18] and Noller [19,20], and more recently joined by Penny *et al. *[21]. Noller [19] has proposed that the ribosome evolved from "smaller functional units capable of carrying out the different translational steps such as peptidyl transfer, decoding, and so on", similar to the view of Penny and co-workers, who have stated, "It is possible that the several active sites of modern ribosomes evolved as separate ribozymes" [4]. Of particular interest in relation to the current proposal, de Duve has suggested that coded protein synthesis arose from a noncoded system in which selection was on the basis of *efficient *peptide synthesis, and that proto-mRNA originally played a *structural *role, immobilizing pairs of proto-tRNAs on an ancestral peptidyl transferase [14-16]; in a recent paper on the origin of introns, Penny *et al. *define the role of the first mRNA in similar terms [21].

In contrast, Wolf and Koonin [1] have proposed that it was the ancestral small ribosomal subunit RNA that first evolved to stabilize the binding of proto-tRNAs to the ancestral peptidyl transferase. However, the demonstration that, "a tRNA bound to the P site of *non-programmed *70S ribosomes [*i.e. *in the absence of mRNA] contacts predominantly the 50S, as opposed to the 30S subunit, indicating that codon-anticodon interaction at the P site is a prerequisite for 30S binding [italics in the original]" [22] argues that the mRNA-tRNA interaction is the more ancestral. Wolf and Koonin have suggested that the first proto-mRNAs arose as part of the small ribosomal subunit RNA, only later becoming discrete entities (this is similar to the idea of Maizels and Weiner [8]). The opposing view is that that mRNA was a diffusible element from the outset, with Penny and colleagues, for example, arguing that mRNA originally evolved from nonfunctional RNA interspersed between functional RNA genes, as part of their "introns first" theory [4], and Crick and co-workers suggesting that mRNA could have been formed initially "*using the anticodon loops of the existing tRNA's *[sic]* molecules *as partial templates [italics in the original]" [23].

## Presentation of the hypothesis

In presenting this model, we take as a starting premise this statement from Wolf and Koonin (2007): "Our main guide in constructing the models is the Darwinian Continuity Principle whereby a scenario for the evolution of a complex system must consist of plausible elementary steps, each conferring a distinct advantage on the evolving ensemble of genetic elements. Evolution of the translation system is envisaged to occur in a compartmentalized ensemble of replicating, co-selected RNA segments, i.e., in a RNA world containing ribozymes with versatile activities". In what follows we seek to demonstrate selective advantage at each step, and, in line with the Continuity Principle, a continuous pathway where information is preserved from the ancestral system to the present. As logic dictates, the evolution of coded peptide synthesis must have occurred in the absence of complex proteins.

### Initial selection for noncoded protein synthesis

Schimmel has proposed an ancestral system of noncoded protein synthesis utilizing hairpin precursors of tRNA [17,18]. Prior to the existence of complex polypeptides, specific aminoacylation would have been catalyzed by the ribozyme predecessors of contemporary protein aminoacyl-tRNA synthetases. Co-existing with these ribozymes was an RNA fragment containing the peptidyl transferase, the ancestral large ribosomal subunit, which functioned as a specific ribozyme promoting the synthesis of short peptides. The evolution of peptidyl transferase activity may have been relatively straightforward, as the primary function of the contemporary peptidyl transfer centre of the large ribosomal subunit appears to be the positioning of the two 3' CCA termini of tRNA through a network of hydrogen bonds [24]. This process would have produced only peptides with random and perhaps repetitive sequences. However, new enzymatic activities could have been possible even in such a primitive context, deduced from what is known today of functions of sequences of repetitive amino acids:

1. Short tandem repeats rich in glycine are able to bind Cu^2+ ^ions [25].

2. A polyglycine-Cu^2+ ^complex exhibits superoxide dismutase activity [26].

Interestingly, Cu^2+ ^cleaves the bond between the aminoacyl-tRNA bond of aminoacyl- but not peptidyl-tRNA [27], suggesting the possibility, if Cu^2+ ^were present in the RNA world environment, of a positive-feedback mechanism in which polyglycine was able to sequester Cu^2+ ^ions, thus enhancing its own synthesis. Additionally, these and other like peptides might have interacted with existing catalytic RNA domains enhancing and developing ribozymal catalysis [4,20,28]. Lastly, polyglycine may have formed stabilizing interactions with the ancestral peptidyl transferase ribozyme [19]: glycine is a well-known 'helix-breaker' that is commonly found in unstructured protein tail and loop regions, such as the internal loop of ribosomal protein L11-C76 which is disordered in the free protein but becomes ordered in its association with rRNA [29].

### Origin of mRNA as stability-enhancer

**Figure 1:**
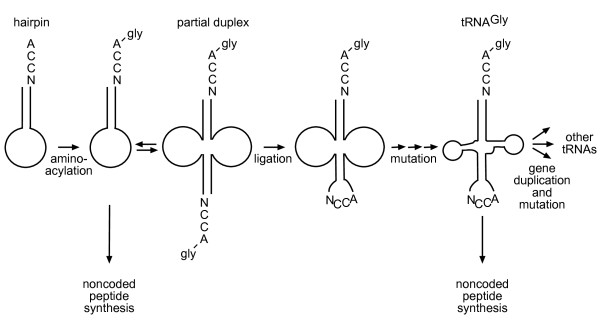
**Proposed hairpin duplication origin of tRNA, based on Di Giulio **[31-33]. RNA hairpin *(left) *was specifically aminoacylated with glycine, enabling it to participate in noncoded peptide synthesis. The hairpin monomer was in equilibrium with the partial duplex *(middle)*, which underwent ligation to form a covalently joined molecule possessing an anticodon loop with the anticodon derived from the 3'-terminal CCA sequence of the upstream hairpin. Mutations produced the first tRNA^Gly ^*(far right)*, also a substrate for noncoded protein synthesis. Subsequent gene duplication and mutation led to a proliferation of tRNA molecules with different amino acid specificities.

Based on the theories of Di Giulio [30-32] and experimental work by Schimmel [33], we have previously proposed on the basis of a highly conserved anticodon loop sequence containing CCA in contemporary tRNAs^Gly ^that the first proto-tRNA (specific for glycine) was formed by the ligation of two RNA hairpins, with subsequent mutations to form the familiar cloverleaf structure [34] (Figure 1). Possessing the same 3'-end structure as its hairpin precursors, this proto-tRNA was similarly aminoacylated and was a substrate for peptidyl transfer catalyzed by the ancestral ribozymes. Subsequently, tRNA^Gly ^gave rise to other tRNAs by a process of duplication and mutation, similar to the concept of Wolf and Koonin [1] (Figure 1). A critical new feature of the tRNA molecule was the central anticodon loop, formed by the head-to-tail ligation of the 3' and 5' ends of the respective hairpins.

**Figure 2:**
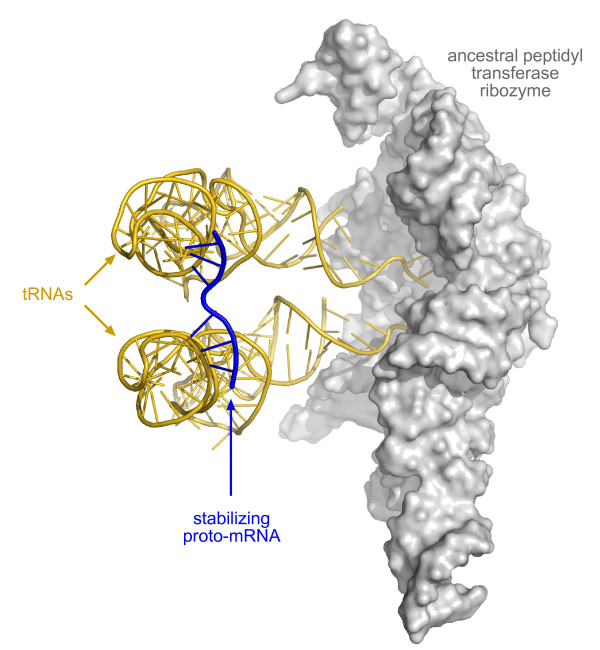
**Our proposal for the origin of coded protein synthesis**. A depiction of the ancestral peptidyl transferase ribozyme as proposed by Bokov and Steinberg [9] with two tRNAs and a serendipitous proto-mRNA binding partner bound to the two tRNA anticodon loops. Adapted from the PDB files of the *T. thermophilus *70S ribosome (with tRNAs and mRNA) taken from Voorhees *et al. *[98]. PDB files rendered using MacPyMol [99].

Accepting that tRNAs derived from hairpins, we propose that the first proto-mRNAs arose as their serendipitous binding partners, forming complementary base pair interactions with the anticodon loops of pairs of tRNAs held in juxtaposition for peptide synthesis by the ancestral peptidyl transferase ribozyme (Figure 2). The proto-mRNA would act as a tether to immobilize the two tRNAs, and with the decreased entropy, enable the 3' CCA termini to be better positioned for peptidyl transfer. Template-independent peptide synthesis of peptides of up to seven residues (indicative of noncoded protein synthesis) was demonstrated by Spirin and associates on contemporary ribosomes with lysyl-tRNA as a substrate in the absence of a directing mRNA [35]. Previously, Monro [36] and, more recently, Wohlgemuth *et al. *[37] have demonstrated the ability of the isolated 50S ribosomal subunit to catalyse peptide synthesis in the absence of mRNA, and Wohlgemuth *et al. *have shown this occurs at the same rate as when catalysed by the complete ribosome. Significantly, Monro [36] detected the synthesis of short peptides using full-length tRNAs as substrates, strongly suggesting these occupied both A and P sites.

Experiments by Jonák and Rychlík [38] demonstrated that binding of oligolysyl-tRNA (with anticodon U*UU, where U* is modified) to the isolated *E. coli *50S ribosomal subunit P site is enhanced approximately two-fold by cognate poly A (but not poly U or poly (U, C)). Similarly, Gnirke and Nierhaus [39] have demonstrated a two-fold enhancement in the binding of deacylated tRNA^Phe ^to the 50S subunit E site in the presence of the cognate poly U (but not poly A). Of interest, Tate and colleagues demonstrated a similar degree of binding enhancement of the protein termination factor RF-2 to the 50S subunit A site in the presence of the cognate termination codon UAA [40]. Although in these examples single site binding to the 50S subunit is highlighted, we would suggest that such binding was possible originally with *pairs *of tRNAs positioned in the adjacent A and P sites, thereby enhancing both the binding of the pair to the ancestral peptidyl transferase and the rate of peptide synthesis. The enhancement in RF-2 binding in the presence of the UAA codon argues that the enhancement is due to the transmittance of a conformational change in the protein from the codon-binding site to the part of the molecule that interacts with the 50S subunit. In the experiments utilizing tRNAs, the poly A and poly U respectively could be regarded as the equivalent of our proposed ancestral stability-enhancing RNAs that were able to base pair with tRNA anticodon loops. Although these experiments were done using 50S subunits containing a full complement of ribosomal proteins, recent structural studies have shown the main contacts for tRNA even on the modern ribosome are with rRNA moieties [41]. These findings support the hypothesis that a complementary RNA sequence (proto-mRNA) could have played a structural role enhancing binding of the first tRNAs to an ancestral peptidyl transferase ribozyme and the rate of peptide synthesis even though the prototype of the contemporary small ribosomal subunit RNA, responsible for coding, was missing.

Subsequent to our developing this model we became aware that a similar concept had been discussed briefly by Christian de Duve in his 1991 book, *Blueprint for a Cell*:

"If the protein-synthesizing machinery first developed without an informational element, how did this element enter the system? In addressing this question, we must remember that, in living organisms today, mRNAs do not only serve to dictate the sequence of amino acid assembly. They also play an essential role in the strategic positioning of aminoacyl-tRNA and peptidyl-tRNA complexes on the surface of ribosomes. It is thus conceivable that *the conformational function preceded the informational one *and that it was, in fact, instrumental in bringing about the latter. Proto-mRNAs could have entered the system as structural adjuncts of the peptide-synthesizing machinery [italics added]" [14].

### The takeover of peptide synthesis by tRNA

In our earlier paper [34] we suggested that the first tRNA(s) might have evolved in an environment of up to eleven specifically-aminoacylated hairpins [24] that were the original substrates for noncoded peptide synthesis. The relative strength of binding of such hairpins and tRNAs to the ancestral peptidyl transferase ribozyme is not known, but the small size of the proposed ancestral peptidyl transferase implies fewer intermolecular interactions than occur on the modern ribosome. However, Schimmel and co-workers have demonstrated a similar rate of peptide synthesis for aminoacylated hairpins and tRNA by the isolated 50S ribosomal subunit [42], indicating the two may have similar binding strengths. If this were true for the ancestral peptidyl transferase ribozyme, the enhancement in binding brought about by serendipitous proto-mRNA binding partners may have led to the eventual replacement of hairpins by tRNA for peptide synthesis. Di Giulio [33] has presented a scenario in which a mixed population of aminoacylated RNA species (including hairpins and cloverleafs) were simultaneously substrates for peptide synthesis.

### Origin of coding

**Figure 3:**
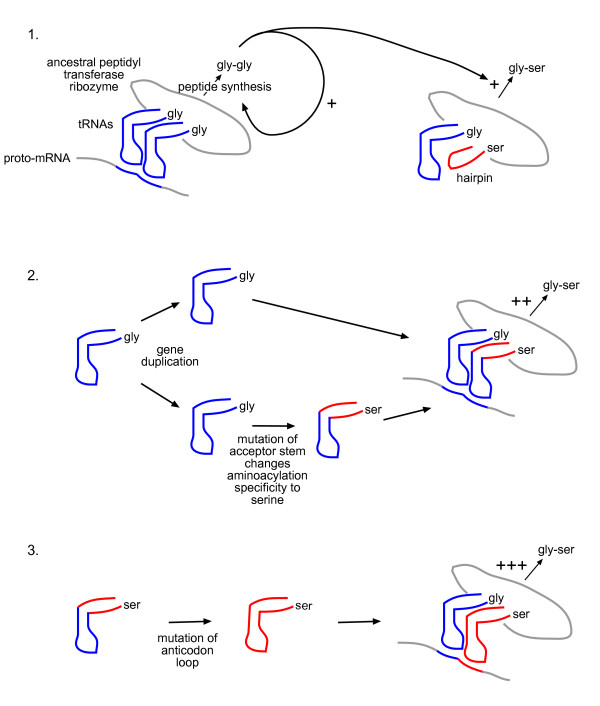
**A detailed scenario for the origin of genetic coding**. 1. Synthesis of gly-gly peptide enhanced by a proto-mRNA complementary to the anticodon loops of tRNA^Gly^, sets up positive feedback loop to further enhance gly-gly synthesis from two tRNAs^Gly^, plus noncoded synthesis of gly-ser from tRNA^Gly ^and the hairpin specific for serine, respectively. 2. tRNA^Gly ^undergoes gene duplication, with one copy undergoing mutation of the acceptor stem to produce a tRNA with aminoacylation specificity for serine (ser). As a result, the synthesis of gly-ser is enhanced by the same proto-mRNA that enhances the synthesis of gly-gly. 3. Selection occurs for mutation of the anticodon loop of proto-tRNA^Ser ^so that the synthesis of gly-ser is *specifically *enhanced. In this way, each new amino acid incorporated into the genetic code is specified by a different sequence (codon). Complementary proto-mRNA and tRNA anticodon loop sequences are represented by the same colour.

The above considerations provide a plausible pathway for the evolution of genetic coding that is outlined below and in Figure 3. Our aim in presenting it is to set forth some of the general principles that we believe may have guided the process, although for the detail the model will undoubtedly need further refinement and experimental validation.

Initially, we assume the presence of a small number of specifically-aminoacylated RNA hairpins (or other RNAs, see below) giving rise to a number of homo- or hetero-dipeptides. If we make the assumption that only two of these were useful, gly-gly and gly-ser, then these two peptides produced by a noncoded process had their production under positive selection because of a net benefit to the compartment or cell.

The original aminoacylated RNAs, described up to this point as 'hairpins', may have actually constituted a mixed population of molecules, having in common only the presence of an acceptor stem-like helix (containing nucleotides of the operational RNA code conferring specificity of aminoacylation) and a 3' CCA terminus allowing specific aminoacylation. (As Schimmel and colleagues have noted, some may have been larger than contemporary tRNA [43], as a number of viral RNAs contain a 3' tRNA-like structure that is specifically aminoacylated *in vivo*).

At this stage the appearance of the first tRNA^Gly ^by duplication and ligation of the glycylated hairpin would have occurred (Figure 1). Subsequent gene duplication of this first tRNA^Gly^, with mutation of nucleotides comprising the operational RNA code in the acceptor stem of one of the copies, would have altered the specificity of aminoacylation by the RNA synthetase, for example from glycine to serine. The production of this new mutant tRNA species would facilitate the synthesis of a beneficial peptide gly-ser, enhanced by the same proto-mRNA that enhanced the synthesis of gly-gly, as both tRNAs would at this stage share the same anticodon. The lack of specificity of the proto-mRNA, however, would also have enhanced the production of ser-gly and ser-ser, perhaps with deleterious effects on the cell. Selection for increased *specificity *would be favoured so that the synthesis of only the useful peptides was increased. If each new amino acid incorporated into the nascent coding system (and its corresponding mutant tRNA) eventually became specified by a different sequence (in other words, by a *different codon*), then each combination of tRNAs would have bound a unique complementary proto-mRNA, giving a one-to-one relationship between proto-mRNA and peptide. This advantage could have driven the evolution of coding specificity - each codon sequence interacted with only a single tRNA, coding for only a single amino acid (Figure 3). De Duve [14] has said the following about this stage:

"Even if precise matching between amino acids and proto-anticodons did not exist initially, its progressive appearance would, predictably, be favoured by natural selection. Consider that we are dealing with a random peptide synthesizer of which there are many copies situated in distinct, competing entities. Mutations of the RNAs involved create the diversity on which selection acts, but within stringent constraints. First, only mutations that respect the topological factors just defined will be tolerated, as others disrupt the machinery. Furthermore, the critical mutations will be those that affect the proto-tRNAs rather than those that affect proto-mRNAs, as it is not yet the quality of the messages that counts, but that of the parts of the synthetic machinery. In such a context, units possessing unambiguous proto-tRNAs will have a manifest advantage over those that have proto-tRNAs associating the same proto-anticodon with different amino acids, the advantage being the possibility of faithful reproduction of a message. In other words, *from the moment translation *[=coded protein synthesis] *became mechanistically possible, its emergence was obligatory *[italics added]".

One constraint on the first anticodon sequences was that they must form stable anticodon-codon interactions in the absence of modified anticodon loop nucleotides, if, as suggested by Crick [44], there was no post-transcriptional modification of tRNA nucleotides at this early stage. Then anticodon sequences (and their complementary codons) with a high G+C content would have an advantage; this may be the reason the first tRNA (tRNA^Gly^) possessed an NCC anticodon.

In overview, the principle selective pressure was for the production of novel useful peptides enhancing biological function, and this in turn resulted in selection for the diversification of anticodon and proto-mRNA sequences. Wolf and Koonin have expressed a parallel concept as part of their proposal for the origin of protein synthesis [1].

### Origin of translocation

Maizels and Weiner [8] have suggested that translocation may have arisen from a slight preference of one of the two tRNA binding sites for peptidyl-tRNA, which meant that, following the peptidyl transfer reaction, the resultant peptidyl-tRNA would slip into the more stable site, displacing the (now) deacylated tRNA, perhaps accompanied by a conformational change in the ancestral peptidyl transferase ribozyme. Something similar occurs on the contemporary ribosome, with the newly peptidylated 3' terminus of the A site tRNA moving spontaneously into the P site following peptidyl transfer [45] (although movement in the opposite direction is not precluded [46,47]). That the whole ribosome possesses an intrinsic ability to undergo translocation is demonstrated by its ability to form peptides in the absence of EF-G and GTP (and EF-Tu) [48-50]. Yonath and associates suggest that translocation is inherent in the structure of the ancestral core of the large ribosomal subunit RNA: "by encircling the PTC [peptidyl transfer centre] it confines the void required for the motions involved in the translocation of the tRNA 3' end, which, in turn, is necessary for the successive peptide bond formations, enabling the amino acid polymerase activity of the ribosome" [12]. If there already existed a weak binding site for the displaced tRNA on the ancestral peptidyl transferase (an ancestral E or exit site), the proto-mRNA may have remained bound to the anticodons of the two tRNAs long enough for the selection of a new (aminoacylated) tRNA for the now empty A site. Wilson and Nierhaus [51] have proposed that the E site is ancestral on the basis of its conservation across all three kingdoms, and they identify three highly conserved nucleotides critical for the binding of the CCA end of the E site tRNA. Intriguingly, these three large subunit nucleotides lie within 40 nucleotides of Bokov and Steinberg's [9] proposed ancestral peptidyl transferase sequence, although, according to their deconstruction of the ribosome, within a region that they predicted was added considerably later to the expanding large ribosomal subunit RNA structure. This implies the structures responsible for release of the deacylated E-site tRNA arose relatively late in the development of the contemporary ribosome. It has been argued that the E site tRNA maintains an anticodon-codon interaction with the mRNA on the contemporary ribosome, with a tRNA cognate to the E site codon able to displace a radio-labelled E site tRNA, while near and noncognate tRNAs are not [52]; recent structural analysis appears to offer some support for an anticodon-codon interaction [53]. When an ancestral E site interaction became possible it would have provided a platform for translocation, giving the ancestral peptidyl transferase ribozyme the ability to utilize longer sequences of stabilising proto-mRNAs productively, the forerunner of protein synthesis as we know it today.

Translocation provides an evolutionary route to coding, even in the absence of the small ribosomal subunit. While in the first instance merely passively binding to the anticodon loops of pairs of tRNAs in the A and P sites, upon movement of the tRNA CCA-termini from the A to P and P to E sites (along with the proto-mRNA), the downstream codon of the proto-mRNA might well have played a role in favouring the selection of the tRNA entering the newly vacated A site. Another way of viewing the experiments of Jonák and Rychlík [38] and Gnirke and Nierhaus [39] that demonstrate stimulation of binding of various tRNA species to the isolated large ribosomal subunit by cognate single-stranded RNAs, is that they demonstrate a form of coding by the large ribosomal subunit. A comparison with the small subunit is instructive. The presence of poly A similarly enhances the binding of oligolysyl-tRNA to this subunit [38]. However, in contrast to the large subunit, non-cognate RNAs cause a *decrease *in binding of oligolysyl-tRNA to the small subunit, reminding us that in conjunction with the small subunit the polynucleotide is playing a selective role in the interaction. As previously discussed, this idea is supported by the demonstration [22] that, "a tRNA bound to the P site of *non-programmed *70S ribosomes [*i.e. *in the absence of mRNA] contacts predominantly the 50S, as opposed to the 30S subunit, indicating that codon-anticodon interaction at the P site is a prerequisite for 30S binding [italics in the original]."

### Origin of the small ribosomal subunit

**Figure 4:**
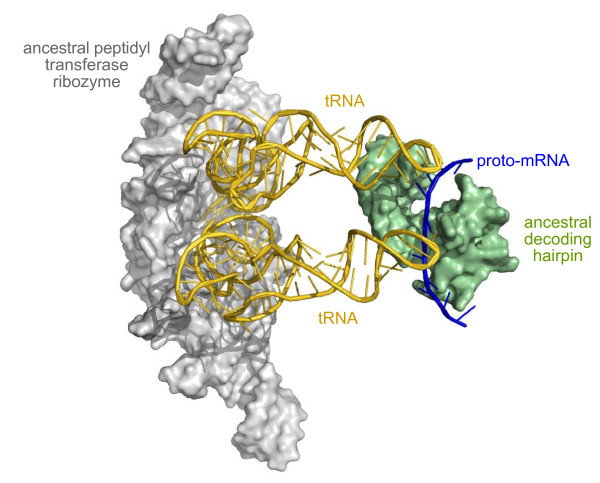
**The origin of the small ribosomal subunit as an RNA hairpin acting *in trans***. A depiction of the proposed decoding hairpin, possibly ancestral to the small ribosomal subunit RNA [54], interacting with tRNAs in the ancestral A and P sites of the ancestral peptidyl transferase ribozyme [9], and a serendipitous proto-mRNA binding partner bound to the tRNA anticodon loops. Note: this view is from the opposite side of the complex to that shown in Figure 2. Adapted from the PDB files of the *T. thermophilus *70S ribosome (with tRNAs and mRNA) taken from Voorhees *et al. *[98]. PDB files rendered using MacPyMol [99].

The small subunit RNA was a later addition to the cohort of RNAs and added fine control over the interaction between tRNA and mRNA so that the fidelity of peptide synthesis was increased. According to Bokov and Steinberg's [9] deconstruction of the large subunit, regions that form interactions with the small subunit occurred in the second stage of the evolution of the large subunit. Although the decoding function of the small ribosomal subunit RNA is made up of a number of segments that are widely separated [7], a 49-nucleotide hairpin comprising part of the decoding site at the 3'-end of the small ribosomal subunit RNA binds both poly U and the tRNA^Phe ^anticodon stem/loop in a similar fashion to the entire small subunit, suggesting such a hairpin-type structure could be an ancestral decoding fragment [54]. This hairpin contains the two nucleotides C1401 and G1402 (*E. coli *numbering) that complex a Mg^2+ ^ion and mark the border between the A and P site codons, thought to be important for maintaining the reading frame and preventing slippage [55]. It also contains the two mobile nucleotides A1492 and A1493 that proofread the anticodon-codon helix [56]. These two nucleotides may have originally functioned in isolation to other elements contributing to decoding in the contemporary ribosome, such as G530, almost 1,000 nucleotides distant in the primary sequence. Intriguingly, there is evidence from the ribosome crystal structure that, if the G530 interaction were missing, A1492 may be able to proofread the second codon position on its own, albeit with lower fidelity, as the ribosomal decoding site can apparently accommodate a G-U base pair at the second position if either A1492 or G530 is utilized, but not with both [57]. Such a decoding hairpin could have originally functioned *in trans *[4], interacting solely with the tRNA-proto-mRNA complex (Figure 4). As discussed above, its acquisition as an intrinsic component of the ribosomal complex and specific interactions with the large subunit RNA would be a later development.

### Summary of the model

The model can be summarized in ten progressive steps. These are listed below with supporting evidence from the literature.

**Figure 5:**
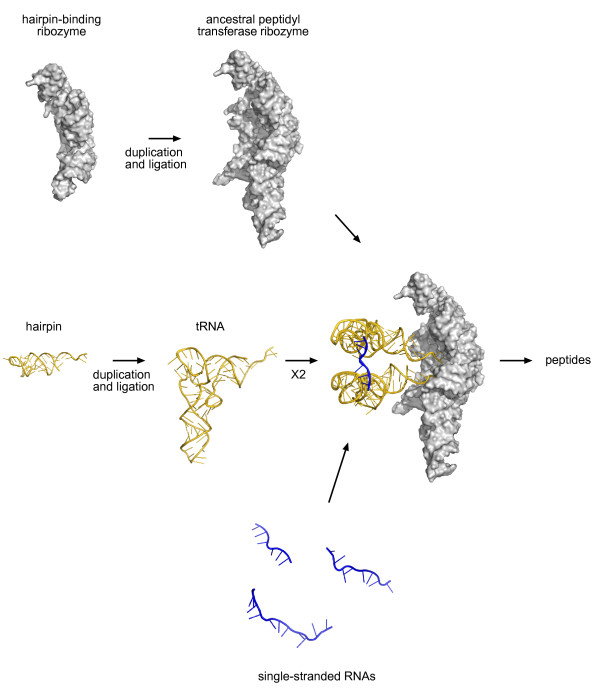
**A duplication-ligation origin of coded protein synthesis**. The origin of coded protein synthesis from the duplication of a hairpin-binding ribozyme [8] to form the proposed ancestral peptidyl transferase ribozyme [9] and the duplication of a hairpin possessing a 3'-terminal CCA [30-32,34] to form the first tRNA. Serendipitous binding of a single-stranded RNA (proto-mRNA) complementary to the tRNA anticodon loops enhanced the binding and positioning of the two tRNAs on the peptidyl transferase, and thereby, the rate of peptide synthesis. Adapted from the PDB files of the *T. thermophilus *70S ribosome (with tRNAs and mRNA) taken from Voorhees *et al. *[98]. PDB files rendered using MacPyMol [99].

(i) *Selection of ribozymes able to aminoacylate a variety of RNA substrates with 3' CCA termini, perhaps related to a role in the replication of genomic RNA*. That this is feasible is supported by the demonstration that self-aminoacylation and the ability to aminoacylate *in trans *are some of the most easily selected RNA functions *in vitro*. For example, a 114-nucleotide ribozyme capable of activating amino acids by catalysing the formation of aminoacyl guanylates (chemically similar to aminoacyl adenylates used universally in biological systems) [58] and a 45-nucleotide ribozyme capable of aminoacylating tRNAs *in trans *[59] have been selected *in vitro *from random populations of RNAs. Amazingly, Yarus and colleagues have very recently demonstrated that a tiny RNA of just five nucleotides can catalyse the aminoacylation of a partly complementary even smaller four nucleotide RNA using the naturally occurring substrate phenylalanine adenylate [60].

(ii) *Serendipitous production of an ancestral peptidyl transferase by a gene duplication and ligation (perhaps of an aminoacyl-RNA synthetase ribozyme), enabling the resulting ribozyme to bind two RNAs with aminoacylated 3' CCA termini, and to promote noncoded peptide synthesis*. Weiner and Maizels [61] have proposed that the first ribosome arose from the gene duplication of a tRNA-binding ribozyme - perhaps an aminoacyl-tRNA synthetase - that created two tRNA binding sites on the same molecule. Bokov and Steinberg's [9] deconstruction of the large ribosomal subunit RNA supports the evolution of the proto-ribosome from the duplication of a fragment of about 110 nucleotides, and Yonath and co-workers have shown that the ribose-phosphate backbones of the A and P sites of the PTC map onto each other extremely closely, suggesting a duplication-ligation origin [12].

(iii) *Generation of a proto-tRNA by duplication-ligation of a hairpin with a specifically-aminoacylated 3' CCA terminus able to participate in noncoded peptide synthesis*. This scenario is supported by the analysis of contemporary tRNAs^Gly ^demonstrating a highly conserved anticodon loop CCA sequence [34] and previous statistical analyses of tRNA molecules by Di Giulio and colleagues arguing for a duplication-ligation origin of tRNA [31-33,62]. Incidentally, the idea that the ancestral peptidyl transferase ribozyme and tRNA both arose by a duplication-ligation event suggests that this may have been a general mechanism for the origin of components of the RNA world [63] (Figure 5).

(iv) *The recruitment of single-stranded RNAs (or RNAs with single-stranded regions) as binding partners to the anticodon loops of tRNA pairs*. This is supported by the ability of single-stranded RNAs to bind to the tRNA anticodon loop free in solution (see the next section for discussion and references).

(v) *Enhancement of the selection of tRNAs as preferred substrates for the peptidyl transferase due to their interaction with single-stranded RNAs (proto-mRNAs), giving rise to a binding enhancement and a consequential rate enhancement of peptide synthesis*. This concept is supported by isolated experiments demonstrating enhanced binding of oligolysyl-tRNA to the P site [38], deacylated tRNA^Phe ^to the E site [39] and the protein termination factor RF-2 to the A site [40] of the isolated 50S ribosomal subunit, dependent upon the presence of the cognate RNA or codon.

(vi) *Synthesis of a greater diversity of beneficial peptides by selection for mutations in the acceptor stem of tRNA, altering the specificity of aminoacylation*. This is supported by the demonstration that a single C70U mutation in the acceptor stem of *E. coli *tRNA^Lys ^results in its aminoacylation with alanine [64].

(vii) *Enhanced specificity for the synthesis of particular peptide products by selection for mutation of the anticodons of these mutant tRNAs, leading to new unique proto-mRNA sequences complementary to the new anticodon loop sequences*. This step is supported in contemporary biology by the existence of suppressor tRNAs that possess altered anticodons complementary to termination codons, that insert the amino acid corresponding to the original tRNA at these positions [65].

(viii) *Synthesis of longer peptides with the emergence of an E site on the ancestral peptidyl transferase*. The relatively early evolution of the E site as the ribozyme expanded is supported by its conservation across all three kingdoms [51] although the analysis of Bokov and Steinberg did not support this conclusion [9].

(ix) *Enhancing the fidelity of protein synthesis by evolution of a short decoding hairpin (the ancestral small ribosomal subunit RNA, functioning *in trans), *to enforce the genetic coding rules*. This is supported by the demonstration that a 49-nucleotide hairpin comprising part of the decoding site of the small ribosomal subunit RNA binds both poly U and the tRNA^Phe ^anticodon stem/loop in a similar fashion to the entire small subunit, suggesting such a hairpin-type structure could be an ancestral decoding fragment [54].

(x) *Control and fidelity of protein synthesis further evolved from a gradual increase in the sizes and interactions between the ancestral peptidyl transferase and decoding hairpin*.

Arguably, each step in this development would have been selected for the ability to produce reproducibly increasingly more complex (and thus potentially more useful) peptides.

## Discussion

### Evidence for tRNA anticodon interactions in the absence of the ribosome

In contemporary protein synthesis, the tRNA anticodon base pairs with the mRNA codon, but this interaction is also stabilized by interactions with parts of the ribosome that can not have been present at the early stages of its evolution. In the experiments of Jonák and Rychlík [38] and Gnirke and Nierhaus [39] previously discussed, the entire 50S subunit (including ribosomal proteins) was used. In order to assess the feasibility of our theory, it is important to look at interactions that occur with the tRNA anticodon loop outside of the contemporary ribosome and its subunits. What interactions with the anticodon loop can occur free in solution? On the basis of a highly conserved anticodon loop 'signature' we proposed tRNA^Gly ^as the first tRNA [34]. Contemporary tRNA^Gly ^contains on average the fewest post-transcriptionally modified nucleotides (and the lowest proportion of modifications at position 37, immediately downstream of the anticodon, important for stabilizing the anticodon-codon interaction through base stacking [66]), with the unmodified state assumed to be ancestral [44]. Three types of interaction occur between single-stranded RNAs and a hypomodified anticodon loop:

(i) *Oligonucleotide-binding of single-stranded tri, tetra and pentanucleotides with the tRNA anticodon loop*. Most pertinent to the ancestral situation are studies using *E. coli *tRNA^iMet^, whose anticodon loop contains a single post-transcriptional modification at position 32 (a 2'-O methylcytidine), a position which is not thought to have a significant influence on anticodon binding [67]. This tRNA has an unmodified anticodon and an unmodified adenine at position 37, and binds a trinucleotide and tetranucleotide complementary to its anticodon (or anticodon plus adjacent U33) with molar association constants of 1.2 × 10^3 ^and 1.4 × 10^4^, respectively [68]. In the absence of the ribosome, tri, tetra and pentanucleotides bind to the anticodon loop of *E. coli *or yeast tRNA^Phe ^with increasing strength [69-72]. These tRNAs, however, contain a hypermodified nucleotide at position 37, and so probably do not represent the ancestral state [44].

(ii) *Interactions between complementary anticodons of two tRNAs*. Interactions between *E. coli *tRNA^Gly ^(with anticodon U*CC, where U* is modified) and tRNA^Ser ^(with an unmodified GGA anticodon) were found to be highly stable [73]; the sole anticodon loop modification in this interaction is that of U34 in the first (wobble) position of the tRNA^Gly ^anticodon, as indicated. Importantly, neither tRNA has a modified purine at position 37, leading the authors to state that, "the presence of three consecutive purines in the [tRNA^Ser^] anticodon triplet together with the two purines on its 3' side may yield sufficient stability..." [74]. The interaction between wheat tRNA^Gly ^and *E. coli *tRNA^Ala ^(with unmodified GCC and GGC anticodons respectively) is also highly stable [75]. In this case the sole anticodon loop modification is a single 5'-methylation of cytidine at position 38 of the tRNA^Gly ^[76].

(iii) *Anticodon interactions with other RNAs*. The GCC anticodon of *Bacillus subtilis *tRNA^Gly ^interacts with a complementary GGC 'codon' sequence within the eight-nucleotide internal specifier loop of the 5'-UTR of the *Bacillus subtilis glyQS *gene, regulating transcription of the encoded glycyl-tRNA synthetase [77]. Deacylated tRNA^Gly ^prevents termination of transcription (and thus increases synthesis of the glycyl-tRNA synthetase, and thereby its own aminoacylation) by also interacting through its 3' UCCA terminus with a complementary UGGA sequence in the anti-terminator bulge of the mRNA transcript, similar to the twin interactions of the anticodon loop and 3' CCA terminus of tRNA on the ribosome. Although the post-transcriptional sequence of the mature *Bacillus subtilis *tRNA^Gly(GCC) ^is not known, by comparison with other tRNAs of similar sequence [76] it is likely to be minimally modified. Significantly, this interaction has been reproduced *in vitro *using unmodified tRNA^Gly^, suggesting that post-transcriptional modification of the tRNA, if present, is not critical for the interaction [77].

The above data suggest that the interaction between unmodified tRNA anticodon loops and single-stranded RNAs, such as we have proposed for the ancestral tRNA and proto-mRNA, would have been strong enough to allow binding in the absence of the contemporary ribosome. The interaction of a single-stranded RNA with *two *adjacent anticodon loops (such as two tRNAs held in juxtaposition by the ancestral peptidyl transferase ribozyme) would have enhanced such binding. Although Labuda *et al. *[78] observed no ternary complexes formed between yeast tRNA^Phe ^in free motion in solution and the complementary *UUCUUC*U oligonucleotide containing two consecutive codons (in italics), our proposal is that the two tRNAs were fixed in place by their 3' CCA termini to the ancestral peptidyl transferase ribozyme, and so were positioned for such an interaction with the proto-mRNA. As we have previously proposed [34], the advent of the genetic coding interaction may have been due to the ability of the unmodified NCCA sequence to form a stable hydrogen bonding interaction with its complementary sequence, with the strength of G-C base pairs and the ability of the adjacent adenine (A37) to base stack on to the resulting helix important [79].

### Origin of the triplet code

Is it possible to deduce from the biophysical data important elements of the origin of the triplet code? Crick [44] suggested that the size of the triplet codon might have been determined by the width of RNA helices, and the closeness with which two adaptor molecules (tRNAs) could approach each other on adjacent codons; this however, would set a *minimum *rather than absolute size restriction. De Duve [14] has proposed similarly a topological basis for the triplet code, the size of which "ensures an optimal spacing of the partners for efficient aminoacyl or peptidyl exchange". Both Crick and de Duve argue from the Continuity Principle that a change in codon length during evolution would have been impossible, as this would destroy all previously encoded information. However, recent structural data demonstrates that the contemporary ribosome is able to accommodate anticodon loops with an extra nucleotide in the reading of four-nucleotide codons, leading Ramakrishnan and associates to make the comment "it appears that normal triplet pairing is not an absolute constraint of the decoding centre" [80]. Along similar lines, Baranov *et al. *[81] have stated that "the emerging picture of decoding strategies used by different organisms...argue [sic] that non-triplet codes or codes with mixed codon sizes are possible"; further, they have provided evidence from computer modelling that decoding systems with codons larger than three nucleotides evolve spontaneously into mainly triplet decoding systems. Grosjean *et al. *[75] argued on the basis of anticodon-anticodon interactions that the natural tendency of seven-membered RNA loops like the anticodon loop is to interact through the three central nucleotides, for example the anticodon triplet. However, as previously discussed, this does not preclude the ability of tRNA free in solution to interact with tetra and pentanucleotides [69-72]. Because of the close similarity Grosjean *et al. *[75] discovered between anticodon-anticodon interactions and the rules of genetic coding, they hypothesized that one of the functions of the ribosome was to fold mRNA into a loop conformation similar to the anticodon loop. While this has not been shown to be the case, there *is *a 45° kink in the mRNA between the codons in the adjacent A and P sites [41]. In view of all of the above, the rationale for the triplet code is still unclear.

### Origin of the coding principle

How did the genetic coding principle arise? Although Rodin and colleagues have postulated that the genetic code (embodied by the anticodon) and operational RNA code (embedded in the tRNA acceptor stem and governing the specificity of aminoacylation) have a common ancestor [82-84], there is a lack of an obvious relationship between them. Rodin and Rodin [84] themselves have stated, "straightforward analysis failed to uncover any traces of homology in this case". Yarus *et al. *[85] have postulated that the organization of the genetic code originated in specific interactions between amino acids and RNA binding sites. However, Ellington *et al. *[86] have pointed out that understanding how such an association (with the requirement for the amino acid-binding site and coding triplet to be in close proximity) would lead to tRNA, with its widely separated sites for the amino acid and the anticodon, is problematic. Specifically, such a scenario would appear to violate the Continuity Principle.

In contrast, an intrinsic feature of our model is that the genetic code has not descended from a previous code, but rather has arisen *de novo*. Wolf and Koonin have stated, "the origin of translation appears to be truly unique among all innovations in the history of life in that it involves the invention of a basic and highly non-trivial molecular-biology principle, the encoding of amino acid sequences in the sequences of nucleic acid bases via the triplet code. This principle, although simple and elegant once implemented, *is not immediately dictated by any known physics or chemistry *(unlike, say, the Watson-Crick complementarity)" [italics added] [1]. We would agree with Wolf and Koonin in part, namely that the principle of genetic coding, derived from the advent of the tRNA anticodon loop (as a novel binding partner for single-stranded RNAs) held in pairs on the ancestral peptidyl transferase ribozyme, was a non-determined event not dictated by physics or chemistry. By contrast, the complementary base pairing interaction at the heart of genetic coding *is *dictated by chemistry, in fact, *by *Watson-Crick complementarity. In the evolution of coded protein synthesis then, it would appear that the advent of the anticodon loop provided the necessary precondition for development of the genetic code. However, this structural invention occurred in a molecule (RNA) able to carry information. The models of Rodin and Yarus postulate the transfer of information from either a pre-existing code (the operational RNA code) [84] or from an amino acid-RNA binding site interaction [85] to the genetic code. On the contrary, we would argue that with the emergence of the molecular assembly described above, genetic coding arose spontaneously due to the intrinsic chemical properties of RNA as an informational molecule.

### Coded protein synthesis - an irreducibly complex system?

Wolf and Koonin [1] have suggested that the problem of the origin of the translation system is so complicated and involves the interplay of so many factors that, at least at first glance, its occurrence "evokes the scary spectre of irreducible complexity". In contrast, our model would suggest that the evolution of protein synthesis was similar to that of other complex systems. A good example is the vertebrate eye; in this instance, Darwin argued, one is able to plot an evolutionary trajectory from a light-sensitive spot to a fully-fledged eye, where each small step was selected for the "particular advantage it conferred onto the evolving organism" [1]. We would argue similarly that peptide synthesis evolved from a more rudimentary noncoded form, and that the synthesis of increasingly more complex peptides provided the selective advantage for a stepwise evolution of the contemporary translation system (similar to the view expressed by Maizels and Weiner [8]). It appears unnecessary to invoke a reassignment of a selectable function during evolution for the protein synthesis machinery.

## Testing the hypothesis

While it would be ideal to test our hypothesis using only *in vitro *transcribed RNA, in practice this may not be possible, as promising reports of protein synthesis by *in vitro *transcripts of the large ribosomal subunit RNA (as well as the individual domains) of *E. coli *were subsequently retracted [87-91]. The contemporary large subunit RNA domain V that includes the peptidyl transfer centre comprises over 600 nucleotides, and achieving the correct folding and function in the absence of ribosomal proteins today may be extremely difficult [88]. More recently, Anderson *et al. *[92] have attempted unsuccessfully to demonstrate peptide synthesis using a 322-nucleotide construct containing the most conserved regions of the peptidyl transfer centre, very close to the proposed ancestral peptidyl transferase sequence of Bokov and Steinberg [9]. Subsequent *in vitro *selection aimed to enrich for peptidyl transferase activity produced a sequence able to ligate A and P site substrates; the ligated product however did not contain a peptide bond. Moreover, product formation was not sensitive to chloramphenicol, indicating that the active site in the RNA, improved by *in vitro* selection, is different from the classical peptidyl transferase centre [92].

Yonath's group has used small stem-elbow-stem (SES) RNA structures in an attempt to demonstrate peptide synthesis. They report that some sequences form dimers, dependent on sequence and the presence of Mg^2+ ^ions; also that "functional experiments, exploring the peptidyl transferase activity of a large variety of the RNA dimers are in accord with the structural analysis" [93]. However, to date no data from these experiments have been published.

Despite much effort, it has not been possible to produce 50S ribosomal subunits that are able to catalyze peptide synthesis in the complete absence of ribosomal proteins [94]. As discussed above, this may be due to a requirement for ribosomal proteins to achieve the proper folding and function of rRNA in contemporary ribosomes.

The experiments that have demonstrated peptide synthesis in the absence of mRNA and enhanced binding of tRNAs in the presence of cognate oligonucleotides have used the whole 50S subunit, including ribosomal proteins. In view of this, a realistic experimental approach would be as follows:

1. Initially, use the isolated 50S ribosomal subunit together with *in vitro *transcribed tRNAs and complementary oligonucleotides to define the parameters for the stabilization of tRNA binding by single-stranded RNAs and enhancement of peptide synthesis, as per the experiments of Jonák and Rychlík [38] and Monro [36].

2. Repeat these experiments using (a) *in vitro *transcribed 23S rRNA together with a minimal set of ribosomal proteins essential for peptidyl transferase activity [95]; and (b) an *in vitro *transcribed 23S rRNA fragment corresponding to the proposed ancestral peptidyl transferase [9] in the absence of ribosomal proteins.

3. Test the ability of polyglycine (and other peptides composed of proposed evolutionarily 'early' amino acids [96]) to stabilize the RNA elements of the system and either increase the rate of peptide synthesis or allow it to occur in the absence of ribosomal proteins.

4. Investigate whether a proposed ancestral decoding hairpin derived from the small ribosomal subunit RNA [54] (*in vitro *transcribed) is able to function *in trans *to control the specificity of binding of tRNAs to the 50S subunit in the presence of cognate RNA, and thus direct the synthesis of specific peptides.

Success in these experiments would bolster the case for our hypothesis, without necessarily discriminating against the counter theory of a replicase/triplicase origin of the ribosome. Penny has suggested that an experimental proof of the latter theory could be carried out using existing RNA polymerase ribozymes generated by *in vitro *experiments [5]. Experimental proof against the proposal by Wolf and Koonin [1] that the ancestral small subunit RNA rather than proto-mRNA was involved in stabilizing tRNA binding to the ancestral peptidyl transferase would not appear necessary, due to experimental evidence demonstrating that, on the contemporary ribosome at least, the small subunit only binds tRNA at the P site in the presence of mRNA [22]. As previously discussed, this suggests that the interaction between tRNA and mRNA predates the interaction between tRNA and the small ribosomal subunit.

## Implications of the hypothesis

The hypothesis presented here is that proto-mRNAs were firstly serendipitous binding partners to tRNA and acted as enhancers of noncoded protein synthesis. It is an extension of our previous proposal of the origin of the first tRNA by a hairpin ligation [34], and suggests that, against a background of noncoded peptide synthesis utilizing aminoacylated RNAs, the advent of the anticodon loop was critical in providing a novel binding surface for the evolution of the first proto-mRNAs. The increased stability these proto-mRNAs conferred on the binding of proto-tRNA pairs to the ancestral peptidyl transferase evolved into a system of coded protein synthesis. This remarkable development could have occurred quite naturally and unremarkably as the portfolio of tRNAs and their serendipitous binding partners gradually expanded. A coding system had been acquired by stealth!

## Competing interests

The authors declare that they have no competing interests.

## Authors' contributions

HB formulated the hypothesis. WT provided original ideas and played a mentoring role. Both authors discussed ideas and wrote the manuscript.

## Reviewers' reports

**Reviewer 1: **Eugene Koonin, National Center for Biotechnology Information, NIH

In this Hypothesis article, Bernhardt and Tate propose a conceptual solution to the great evolutionary puzzle, the origin of protein coding and translation. The idea is that initially the proto-mRNA played a structural role in facilitating non-templated peptide synthesis rather than an informational role as is the case in modern translation. I find this hypothesis very plausible. Indeed, it is a natural extension of or variation on the detailed scenario for the origin of translation presented by Wolf and Koonin [1]. Furthermore, the authors gamely note that an idea essentially similar to theirs was proposed by De Duve in his 1991 book [14]. Considering these predecessors and the extensive discussion and citation in the present article, the paper of Bernhardt and Tate reads, perhaps, more like a review than a hypothesis in the strict sense. This is not a criticism: the discussion is thoughtful and thorough, and should be appreciated by readers interested in the origin of translation which indeed is central to the origin of modern-type life.

### Authors' response

As noted in our acknowledgements, Koonin and Wolf's 2007 paper in Biology Direct [1] was an inspiration for our model. The theory of a structural role for mRNA preceding its informational one (in the words of de Duve [14]) was our own, and the earlier work by de Duve was only discovered at a relatively late stage during preparation of the manuscript. Surprisingly, although de Duve's ideas were first published almost 20 years ago [14] and have been published on two occasions since then [15,16], there appears to be very little general awareness of them by researchers in the field, as can be seen by an absence of reference to his work in recent reviews of the area [97,1,5]. An exception is the recent paper - "An overview of the introns-first theory" - by Penny and colleagues [21]. However, the origin of coded protein synthesis is not the main focus of the paper, and reference to de Duve's theory is made in a single figure and its accompanying legend without citation. While we acknowledge our hypothesis is not completely novel, we feel our paper presents the concept in a new context, namely within a model for the evolution of coding from noncoded peptide synthesis. The realisation that a coding scheme could develop by stealth was a 'eureka' moment for us. In addition, we have highlighted isolated original work by Jonák and Rychlík [38], Gnirke and Nierhaus [39] and from our own lab [40] that provides experimental support. Lastly, when we discovered de Duve's earlier reference to the idea, we were excited that the central concept has a naturalness that has occurred to more than one person in the field, and one of great stature; in fact, as de Duve [16] notes, " [This theory] corresponds to what is about *the simplest and most straightforward course of events that can be imagined *to account for the development of RNA-dependent protein synthesis. *Most workers who have thought about the question have come up with more or less similar solutions*" [italics added]. The low awareness in the field of de Duve's concept, alluded to in three books over a 14-year period [14-16], perhaps also tells us that as scientists we should be more cognisant of significant books!

**Reviewer 2: **Rob Knight, University of Colorado

In this manuscript, the authors provide a model for the evolution of coded translation based on the concepts of RNA hairpins as handles for amino acids used as coenzymes or in noncoded peptide synthesis, origin of tRNAs via hairpin duplication, and expansion of the genetic code from a primordial repertoire of a few amino acid specificities in the original hairpins and/or tRNAs. They propose based on their earlier work and based on the ability of poly(Gly) to bind Cu2+ and exhibit superoxide dismutase activity that the original selection pressure was to produce poly(Gly) or similar peptides, that this ability was then extended to the ability to make simple copolymers of other amino acids, that proto-mRNAs initially evolved as RNA effectors that assisted in the orientation of hairpins for non-coded peptide formation and only later evolved coding potential.

The problem of how the translation apparatus evolved is an important one, especially because it is important that proposed mechanisms provide some sort of continuity of function (so that each subsequent step is an improvement). As the authors note, the steps in the process they have proposed have largely been proposed before (e.g. by de Duve, by Weiner and Maizels, by Wolf & Koonin, by Yarus, by Knight & Landweber, etc.) so the question becomes (i) whether the pathway is sufficiently interesting to be publishable as a starting point for discussion, and (ii) whether the pathway is sufficiently compelling that we should accept it as a likely account of how the code evolved.

My impression is that the model passes the first test but not the second. As the authors note, one attraction of this model is that several steps are empirically testable, although the experiments are not clearly described in the present version of the manuscript. The manuscript is also relatively long in relation to its news value and could benefit from a substantial reorganization (especially because a lot of what appears in the discussion about different alternative models should really be in the discussion.

I would suggest the following reorganization/refocusing:

- In the introduction, clearly enumerate the competing models (grouping related models into families) and highlight the main differences among them.

- Separate the speculations about the selective pressures that might have driven the original nontemplated peptide synthesis function into one section, rather than repeating variations on this material in several places.

- Present the model earlier: the model and the methods for testing it appear on p 30 and take 2 pages out of the 45 in this version of the manuscript. Since the new model is what is central in this paper, it should appear earlier and then the steps should be justified with citation of specific, appropriate literature.

- In general, shorten both the introduction and the discussion considerably to focus more on the proposals that are directly relevant to this proposal.

- In the discussion, clearly enumerate what experiments you would do in order to test each step of the model, how the outcome of each experiment could discriminate between this model and the alternatives mentioned in the introduction, etc.

### Authors' response

We agree with the reviewer's suggestions and have revised the manuscript accordingly.

- I was surprised not to see the original Yarus DiRT paper, or the Knight and Landweber 2000 piece on alternative models for getting from triplet/site associations to the present, or the original Szathmary paper on coding coenzyme handles, in the reference list although I acknowledge there could be reasonable motivations for omitting these and the reference list is long already.

One additional question I have is whether the Cu2+/superoxide dismutase selection pressure is a reasonable candidate: since these events presumably took place before the oxygenation of the atmosphere, how strong a selective advantage would SOD activity provide? Interestingly, Schwedinger & Rode reported in 1989 that Cu2+ could catalyze peptide bond formation at high temperature/salt (Analytical Sciences 4:411), so the copper binding activity could be more directly relevant to peptide evolution. However, this weakens the argument that Cu2+ sequestration would be necessary (and, given the likely levels of sulfides, what would the Cu concentration have been in the early oceans?)

### Authors' response

We acknowledge the discussion regarding potential interactions of glycine peptides with Cu^2+ ^was not well developed in the original manuscript (see also comments by reviewer 3), and this has been revised. The examples of glycine-Cu^2+ ^chemistry, while interesting from our point of view - having proposed tRNA^Gly ^was the first tRNA [34] - were given to illustrate the possible roles that could be played even by very simple peptides. The 1989 paper by Schwedinger & Rode is indeed interesting, suggesting a possible prebiotic route for the synthesis of short peptides. However, we would argue that such mechanisms were ultimately supplanted by firstly noncoded, and then coded protein synthesis.

I think the model presented here is interesting and hope that the manuscript can be rewritten in such a way as to inspire the empirical testing that it deserves. In particular, the demonstration that a generic proto-mRNA could enhance the rate of nontemplated protein synthesis using aminoacylated hairpins would be an interesting finding.

**Reviewer 3: **Berthold Kastner, Max Planck Institute, Göttingen

Bernhardt and Tate present in their manuscript "The transition from noncoded to coded protein synthesis: did coding mRNAs arise from serendipitous binding partners to paired tRNAs that enhanced peptide synthesis on an ancestral peptidyl transferase?" a very compelling scenario of evolution of coded protein synthesis. In addition, the line-up of the various steps leading from the RNA- to the RNP-world presented at the end of the manuscript is very attractive, as each step could lead to an advantage of the system. For readers from outside the field it might be helpful if this concept is presented in a short version already in the results section. The many possible individual steps of the evolution scenario formulated already previously require thorough discussion that leads to a quite lengthy manuscript. Nevertheless, it might be possible to compact it a bit more. For one of the steps, the first appearance of di-/oligo- peptides, the reviewer sees the focusing on the Cu2+ binding argument for the evolutionary advantage as being rather narrow. As we have very limited perception on the chemical environment that might have existed in the RNA-world "cell", the availability of short peptides could have given the system an advantage for various chemical pathways. It might have even been as simple as the prime benefit of amino acids have been the ability of buffering the pH of the system and di-peptides would have then the advantage of being more confined to the containment (the "cell"). The pH buffer function of proteins is still important in modern life. Then, the coded protein synthesis might have brought about the more specific functions of the peptides and mark the starting point of the RNP world. Still, detailed scenarios of such early stages of evolution remain mostly speculation, but the scenario presented here for evolution of the coded protein synthesis is highly plausible within the framework of current thinking. With the presented new perspectives and the careful discussions the manuscript of Bernhardt and Tate is well suited for publication in Biology Direct.

### Authors' response

As discussed in our response to the last reviewer, the examples of glycine-Cu^2+ ^chemistry were given to illustrate the catalytic possibilities of simple peptides. The reviewer's suggestion of early peptides having a role in the maintenance of pH is an interesting one, and worthy of further investigation.
